# Environmental Physical Activity Cues and Children’s Active vs. Sedentary Recreation

**DOI:** 10.3390/ijerph19031874

**Published:** 2022-02-08

**Authors:** Amanda N. Spitzer, Katrina Oselinsky, Rachel G. Lucas-Thompson, Dan J. Graham

**Affiliations:** 1 Department of Psychology, Colorado State University, Fort Collins, CO 80523, USA; katrina.oselinsky@colostate.edu (K.O.); dan.graham@colostate.edu (D.J.G.); 2 Department of Human Development and Family Studies, Colorado State University, Fort Collins, CO 80523, USA; lucas-thompson.rachel.graham@colostate.edu; 3 Department of Community and Behavioral Health, Colorado School of Public Health, Fort Collins, CO 80523, USA

**Keywords:** physical activity, children, physical environment, priming, active play, signage

## Abstract

Physical activity (PA) benefits health, and intensive environmental modifications can increase children’s PA. Research has not yet addressed if subtle environmental cues, such as posters depicting PA, increase child PA. In the current study, it was hypothesized that children exposed to active posters (vs. nature posters) would spend a larger proportion of free play time engaging with active toys (relative to sedentary toys). Participants were randomly assigned to one of two conditions in which posters on a laboratory wall depicted 1. People being active, or 2. Nature scenes. Children aged 5–10 years (N = 175) could play with up to eight toys (four active, four sedentary) while parents completed study-related surveys. The proportion of playtime that was active was compared between groups. Poster type did not have a significant effect on proportion of active playtime. Previous environmental interventions that increase children’s PA have done so through enhancing access to active opportunities, rather than via signage. It is possible that poster interventions such as this may not influence children’s PA, or perhaps other types of cues would have been more effective. Future research should investigate subtle environmental cues that match both the target audience and the accessible PA options (e.g., posters depicting children playing with available active toys) and explore other low-investment environmental modifications to boost children’s PA.

## 1. Introduction

American children are afforded approximately 60 h of discretionary time per week [1]. They fill this time with both active and sedentary behaviors, but much more of this time is spent sedentary [2,3]. It is estimated that 42.5% of children aged 6–11 years perform the recommended 60 min of physical activity (PA) every day [2]. In contrast, about 62% of children in this age group exceed the recommended 120 min or less of TV/video watching per day [3,4], and the average amount of sedentary time per day is 8 h [5]. Choosing to engage in active behaviors during discretionary time benefits children’s physical [6,7,8], cognitive [9,10], and emotional wellbeing [7,8,10,11,12,13]. Within grade-school populations, PA interventions can decrease body mass index (BMI) [6,8], high blood pressure [7], and depressive symptoms [7,13], and can boost positive affect [8,11]. Correlational studies have also linked higher physical fitness with higher cognitive performance and increased volume of subcortical brain structures associated with executive function and memory [9].

Previous research has identified many psychological, social, and environmental influences on child PA [14,15,16,17,18,19,20,21,22,23,24,25,26]. Among psychological correlates of greater PA are greater self-efficacy [14,18,19], fewer perceived barriers [15], and intention to be active [15,19,20]. Social correlates include parental support [14,18], parental PA [16], peer PA [21], and having parents, peers, and siblings watch PA engagements (i.e., attending a child’s sports game) [17].

Environmental influences can also affect children’s behavior [15,18,22,23,24,25,26] and are the focus of the present study. Time spent in outdoor environments, for example, is associated with increased PA [15,18]. Several studies have negatively correlated distance to parks and playgrounds with children’s PA [22,24], particularly parks with fields [26] (i.e., when PA amenities are closer, PA is higher). Furthermore, playground renovation has been linked to increased vigorous PA [23] and increased playground patronage by children [23,25]. Beyond the playground, a review of quantitative literature concerning environmental attributes and children’s PA identified the presence of sidewalks as a consistent correlate of children’s walking and biking behaviors [22]. More broadly, multiuse paths within a child’s neighborhood are associated with higher moderate-to-vigorous PA (MVPA) [26].

Bringing about large-scale environmental changes, such as adding parks and sidewalks to neighborhoods, is costly and not always feasible, but there is some evidence that smaller, less-expensive environmental changes can also influence children’s PA behaviors [27,28,29]. Colorful paint markings on playgrounds have produced increases in PA, especially among older grade-school children [27]. The introduction of movable/recycled materials such as crates and pallets into school playgrounds has resulted in increased active play [28]. In classrooms, the replacement of traditional seated desks with standing desks and stools increases energy expenditure [29]. These small-scale physical environmental changes are consistent with choice architecture theory [30,31] which posits that behaviors can be altered by altering the environment.

A possible way to utilize the environment to prompt children to be more active that requires even less monetary and time investment than those methods presented earlier is through signage. Signage aimed at increasing PA has not been investigated in children, although there is robust research supporting its use in adults [32,33,34,35,36,37,38,39,40,41]. For instance, in adults, signage encouraging PA is associated with elevated MVPA within a park [32]. In addition, signage interventions can increase adult stair use [38,39,40,41]. Increased stair-taking induced by signage can be sustained for at least two years when signage is maintained [42] and can also generalize to unaltered stairs [43]. The present study examines if there is evidence that the PA-increasing effect of relevant signage found in adults could be comparable in child populations by testing if children are more active in the presence of posters depicting people being physically active than posters depicting nature scenes.

Signage is a visual cue, and visual cues have been shown to affect children’s health behaviors; specifically, children’s eating choices can be influenced by the presence of particular visual cues on packaging, such as cartoon characters [44] and illustrations [45].

One avenue by which visual cues can prompt behavior is through priming. Priming occurs when exposure to a stimulus (such as words and images, as on signage) increases the likelihood of a target behavior after exposure to another stimulus (such as the opportunity to act) (see review by Janiszewski and Wyer [46]). For example, religious individuals primed by religious terms or contexts show increased behavior that aligns with their religious views (e.g., prosociality) [47]. This effect is transient as the mechanism of this link is the activation of relevant mental constructs; it follows that the original stimulus must be suggestive of the target behavior. Previous research has demonstrated that priming effects have been documented in children for primes presented over widely varying exposure times (from fractions of a second to many minutes of exposure) [48,49,50]. One example of priming in children is the weapons effect, where aggression is primed by the presence of a weapon [48,49]. Most pertinent to the present study, children’s health-related choice behaviors are responsive to image-based priming, such as healthy eating choices [51]. Of yet, image-based primes, such as posters, have not been researched in the context of children’s PA.

This literature makes it clear that the environment is an influential predictor of PA in children, but the robust influence of signage has only been investigated in adults [32,33,34,35,36,37,38,39,40,41,42,43], not children. In order to address the >50% of school age children [2] that do not meet PA guidelines, research must investigate questions such as whether signage can also promote PA among children. 

The present study sought to determine whether inexpensive environmental cues serve as primes and thereby affect children’s toy use in a randomized laboratory experiment. This study does not compare the effects of small- and large-scale environmental changes; rather, its findings concerning environmental cues offer insight into whether one-time exposure to PA-related signage is effective in promoting PA among child populations.

Specifically, children involved in a larger study had approximately 20 min, while their parents were completing questionnaires, where they were permitted to play with active and/or sedentary toys in the laboratory. Participants were randomly assigned to one of two conditions: active posters (two posters—one depicting swimmers and one depicting runners) or neutral posters (both depicting nature scenes). The posters were all the same size (22” tall × 28” wide) and hung on a wall in a university laboratory where children were allowed to freely choose to play with as many of eight available toys as they would like while their parents completed questionnaires.

Based on previous research demonstrating how even very subtle environmental cues can prompt behavior change [27,32,33,34,35,36,37,38,39,40,41,42,43,46,48,49,51,52,53,54,55,56,57,58,59], we hypothesized that children exposed to the active posters spend a greater proportion of their playtime engaged with active toys (relative to sedentary toys) than children exposed to the nature posters. As in previous research demonstrating downward trajectories in PA as children age [2,18,60,61,62,63,64,65], and greater PA among boys than among girls [14,15,17,18,24,26], we also tested whether younger children and boys would spend a greater proportion of their playtime with active toys, compared with older children and girls, respectively. It was not expected that the effects of the posters would vary based on child age or sex, but this possibility was also tested.

## 2. Materials and Methods

### 2.1. Participants

The participants in this study were N = 175 children aged 5–10 years old (mean age of 7.63 [1.30] years). These children were 53% male/47% female; 86% of the participants were non-Hispanic White, and 14% were Hispanic. Participants came to a university laboratory with one parent as part of a larger study on consumer behavior. 

### 2.2. Procedures

During the portion of the study from which the present data were collected, 8 toys were freely available to children to play with while their parents were at the other end of the laboratory space completing questionnaires. Toy options were 4 active toys designed for children to use while moving around (jump rope, mini bowling set, basketball and hoop, and golf club with balls and hole) and 4 sedentary toys that children use while seated (puzzles, coloring pages, play dough, and magnetic doodle board). Children could choose as many of the 8 available toys as they wanted during the time that their parents were completing questionnaires as part of a larger study. Some children participated in activities that did not involve the provided 8 toys; in order to avoid inconsistencies in coding, only time spent engaging with one of the 8 provided toys was included in analyses. Because the children’s total play times varied somewhat (dependent on how long it took the parents to fill out a survey), proportion variables were created to ensure a consistent unit of measure across all children. To create these proportion variables, the total number of minutes spent playing with any combination of the active toys was divided by the total number of minutes spent playing with the provided toys (active or sedentary). This created the new variable, proportion of playtime that was active, which was used in the following analyses. 

### 2.3. Statistical Analyses

Analyses were conducted in 2021 using R Statistical Software version 3.6.2 (R Core Team, Vienna, Austria). Independent samples *t*-tests were used to determine the effect of poster condition (posters depicting active play vs. posters depicting nature scenes) on children’s proportion of playtime that was active. To probe the relationship between poster exposure, participant age, sex, ethnicity, and the proportion of playtime that was active, multiple linear regression analyses (MLR) with categorical predictors were utilized. No demographic differences were identified between the poster groups in terms of age (Mactive = 7.7 years, Mnature = 7.4 years; *p* = 0.167) and ethnicity (active group: 85% white, nature group: 87% white; *p* = 0.742); however, there was a significant difference in terms of sex with a relatively higher proportion of males and lower proportion of females being randomized to the active-poster condition (60% male, 40% female) relative to the nature-poster condition (46% male, 54% female). To account for the unequal distribution of sex to poster condition, sex was added as a control variable in the MLR models.

A Shapiro Test for normality indicated that the outcome variable (proportion of playtime that was active) did not conform to a normal distribution (S1 *p* < 0.01). Although the aforementioned analyses are robust against deviations from normality for large sample sizes (*n* > 30), we also conducted the nonparametric Mann–Whitney test and used bootstrap confidence intervals to confirm the accuracy of the *t*-tests and MLR results, respectively. 

Previous research [60,61,62,63] indicates that as children age, they tend to engage in less active play, instead opting to participate in more sedentary activities. To test whether this pattern also emerged in the context of a short-term laboratory study, interaction models were run to assess whether age was related to the proportion of playtime that was active, and whether effects of condition varied by age. 

## 3. Results

**Figure 1:**
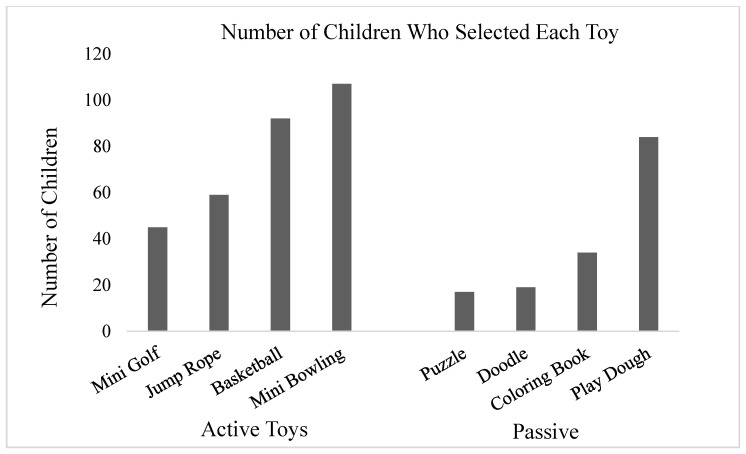
Number of children who selected each toy.

Children chose to play with between zero and eight toys during their approximately 22 min sessions (mean number of toys = 3.04, *SD* = 1.74; mean session length = 21.88, *SD =* 10.77 min). On average, participants played with 1.97 active toys and 1.07 sedentary toys. Overall, 30% of participants opted to only play with active toys and 16% with only sedentary toys. In total, 11 participants refused to play, and 7 participants had missing data resulting in their removal from analyses. As shown in Figure 1, of the eight toys, children most often chose to play with the mini bowling set (*n* = 107) followed by the basketball (*n* = 92) and the play dough (*n* = 84). The toys that were selected the fewest number of times were the doodle board (*n* = 19) and the puzzle (*n* = 17). 

**Figure 2:**
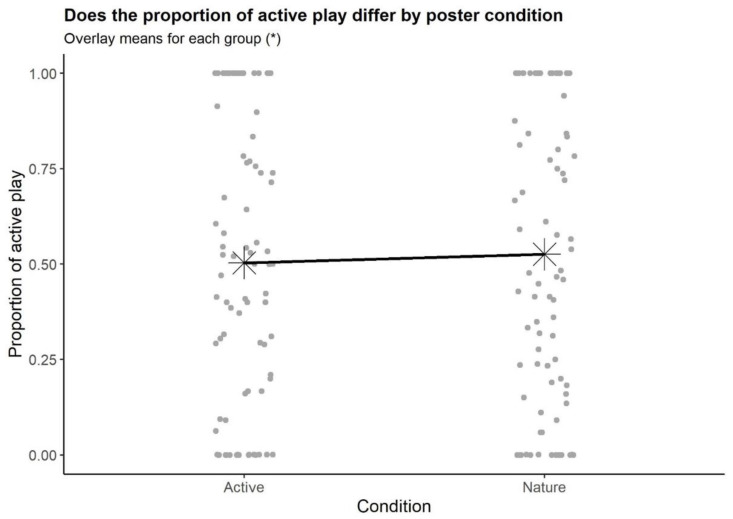
Proportion of playtime that was active by condition. * indicates the mean proportion of active play for participants in that condition.

The results of the *t*-test indicated that poster type did not have a significant effect on the proportion of playtime that was active (54% in the active-posters condition vs. 56% in the nature-posters condition; *p* = 0.722; see Figure 2). Follow-up analyses to adjust for the evident non-normality also indicated no significant differences in proportion of playtime that was active (*p* = 0.761). 

**Figure 3:**
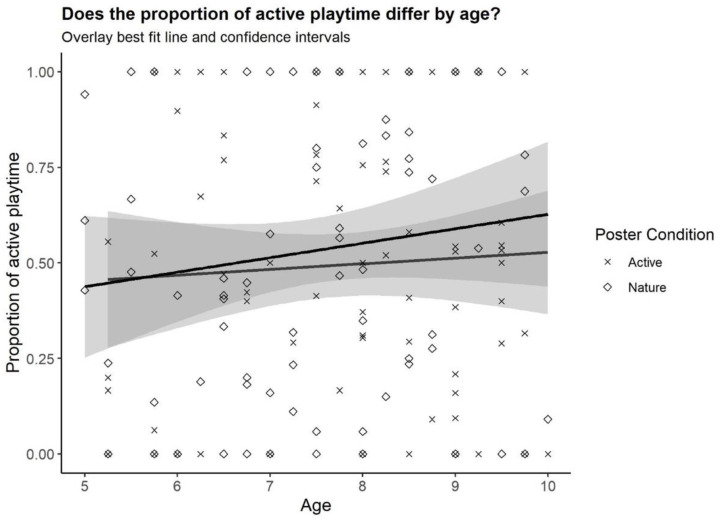
Proportion of playtime that was active by age.

**Figure 4:**
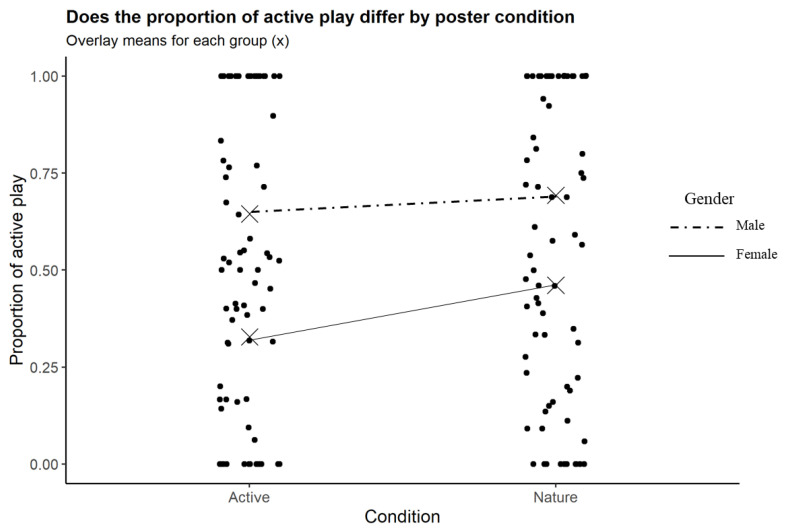
Proportion of playtime that was active grouped by poster condition and participant gender.

Two interaction models were evaluated to examine the differential effect of age and sex on the proportion of playtime that was active (see Table 1 and Table 2). The first moderation model was estimated with age regressed on the categorical indicator of poster type and sex and the poster x age interaction. Sex was significantly different between the groups (*p* < 0.001), indicating that when holding age and poster condition constant, females engaged in approximately 32% less active playtime than males. Additionally, age was significant, demonstrating that for those children who were in the active-poster condition, a one-year increase in age corresponded to a ~6% increase in the proportion of playtime that was active, holding sex constant. Although age was significant in isolation, the interaction between age and poster condition was not statistically significant, indicating there was not a differential effect of age on the proportion of active playtime for those in the active poster condition compared to those in the nature condition (see Figure 3). The results of the second moderation model were the same, and no significant interactions between poster condition and sex were identified (see Table 2 and Figure 4). Finally, in both models, the *R*^2^ value was significant, illustrating that these models explain a significant proportion of the variability (18.5%) in the outcome variable (the proportion of playtime that was active). 

## 4. Discussion

This study was designed to test whether subtle environmental cues (i.e., posters depicting people being physically active in the same location where children chose toys to play with) would encourage children to play with more active toys (compared to children exposed to nature posters). Whereas child food-related behaviors have been shown to be influenced by visual primes [44,45], our results did not show a similar effect for PA behavior. Contrary to our hypotheses, and to choice architecture theory [30,31] and priming research [46,48,49,51,52,53,54,55,56], the results indicated that in this study, children’s proportion of playtime that was active did not differ by poster condition. This suggests that poster interventions such as this one may not influence children’s PA behavior. Of yet, research has only supported the effectiveness of environmental interventions that enhance access to active opportunities, such as introducing new PA resources or restructuring play/learning environments [27,29]; moreover, our results suggest that environmental cues targeting PA through priming may not be effective in children.

It is also possible that no between-condition differences were recorded because our comparison, nature posters, was not as neutral as intended, and participants randomly assigned to this condition were also influenced to be more active than if no posters at all had been present. In research conducted after our choice was made to use nature posters as a comparison group in the present study, Stöckli and colleagues [56] found nature posters to be associated with healthy behaviors (specifically healthy eating in their study, which also used both a nature poster and an activity poster in their priming intervention on snack choices). These authors observed an increase in healthy eating choices after exposure to the nature poster compared to no poster and no difference between the nature and activity poster conditions. Therefore, our “neutral” nature poster condition could have primed participants to engage in active play equally as much as the active poster did. Future research along the lines of what was conducted here could benefit from including a no-poster comparison group to test this possibility that both poster types used in the present study may have promoted greater active play. 

A further possibility is that the expected priming effect of viewing other people being physically active did not translate into greater PA among these participants because of one or more differences between the individuals portrayed in the active posters and the study participants (e.g., the individuals portrayed in the posters were adults, and our study participants were children; the individuals shown in the active posters were running and swimming, whereas the active toys in this study were not directly related to either of the activities shown in the posters). The role of self-similarity in behavioral priming is supported by virtual avatar priming studies [66]. More generally, persuasive messaging is more effective when presented by an ingroup member [67,68,69], particularly when group membership is germane to the topic of messaging [70], which is consistent with self-identity theory [71] and self-categorization theory [72]. It follows that children may not be influenced by messaging concerning leisure activities seemingly presented by the outgroup members in the posters (i.e., adults). With respect to incongruence between the running and swimming primes and the target active play options, research in semantic priming can shed light. Relatedness between stimuli and target words influence the effectiveness of semantic priming, such that a lower relatedness between stimuli and target reduces priming effects [73]. Although our stimuli (running/swimming posters) relate to the target behavior (the active play that is afforded by our provided toys), the degree of relatedness may be too modest for priming to be successful.

Additionally, contrary to existing research [2,18,60,61,62,63,64,65], child age was unrelated to the proportion of playtime that was active. Previous research reporting that PA decreases with age has primarily utilized accelerometer methods, with data collected over a period of days [60,61,62,63,64]. It is possible that age-related decline in PA is driven by a decline in active behaviors that were not measured in our short-term laboratory study but are measured by multiple-day monitoring. For example, if the age-related decline is primarily driven by a change in locomotor movement, such as walking or running, it follows that in our experiment there would be no age-related decline in the proportion of playtime that was active, as our setting, a university laboratory, was not supportive of those activities. Additionally, there is some support for the role of social support in age-related decline in PA [74]. In the setting of our experiment, social factors of age-related decline may have not been present, as most (89.9%) children did not play with a peer. Research staff noted whenever participants engaged in play with another child (e.g., a sibling), which occurred only 16 times. 

The observed sex differences in active play are consistent with previous research [14,15,17,18,24,26]. Multiple reviews have found that boys engage in more PA than girls [14,15,18]. Reimers et al. [75] observed a difference in the pattern of active play between boys and girls at playgrounds: boys disproportionately played sports and games compared to girls, who were more likely to participate in locomotor movement or play on playground equipment. The toys that were available to participants in our study more closely fit the pattern of boys’ play described in Reimers et al. [75], which may further explain the sex differences we observed.

This study had several strengths, including the experimental design involving random assignment of children to the active- and nature-posters conditions, the enrollment of 175 children aged 5–10 years old, and the testing of a subtle environmental priming cue not previously tested in terms of potential effects on type of play. Although children were not required to use the active toys in the active way they were intended to be used, nor the sedentary toys in a sedentary manner, research staff recorded all instances when toys were used in unintended ways, so we were able to code the type of play based on how the toys were used (in an active vs. sedentary manner), and not just which toys were used. While a few children chose to play with a toy in a novel way, there were no instances in which an active toy was used sedentarily, nor in which a sedentary toy was used actively.

The study also had limitations, including the limited range of active and sedentary toys that were available for use and the artificial setting in which children were asked to play, in a university laboratory with a stranger (research assistant) watching. Children may have played differently than they would when in their homes or when they play with peers, instead of by themselves as 89.9% of our participants did.

## 5. Conclusions

The subtle environmental manipulation tested here (i.e., posters showing people being physically active) did not increase short-term PA behavior among children, even though similar small-scale signage manipulations have been found to be effective in adults [32,33,34,35,36,37,38,39,40,41]. Future research could test whether images of active individuals more similar to the target individuals (e.g., similar age, similar activities) or images showing more readily accessible PA options (e.g., children playing with the available active toys) might be more effective in increasing PA in the short- and longer-term. Researchers should also explore other PA-related visual cues to identify which may be more powerful primes toward boosting children’s activity. One possible line of future research stems from embodied cognition theories, which hold that cognition partially emerges from body–environment interactions [76]. It follows that immersive experience may be more effective at altering behavior than viewing poster images; consequently, research may benefit from investigating mixed reality as a possible vessel for PA-related environmental cuing. 

## Figures and Tables

**Table 1:** Linear regression examining the interaction between poster condition and age using the proportion of playtime that was active as the criterion ^1^.

Predictor	*b*	*b* 95% CI [*LL*, *UL*]	*sr* ^2^	*sr*^2^ 95% CI [*LL*, *UL*]	Fit
(Intercept)	0.48 **	[0.29, 0.66]			
Poster Type	0.06	[−0.19, 0.31]	0.00	[−0.01, 0.01]	
Sex	−0.32 **	[−0.44, −0.20]	0.16	[0.05, 0.26]	
Age	0.06 *	[0.00, 0.12]	0.02	[−0.02, 0.07]	
Poster Type *Age	0.02	[−0.07, 0.11]	0.00	[−0.01, 0.01]	
					*R*^2^ = 0.185 **
					95% CI [0.07, 0.28]

^1^ A significant *b*-weight indicates the semipartial correlation is also significant. *b* represents unstandardized regression weights. *sr*^2^ represents the semipartial correlation squared. *LL* and *UL* indicate the lower and upper limits of a confidence interval, respectively. * *p* < 0.05, ** *p* < 0.01.

**Table 2:** Linear regression examining the interaction between poster condition and sex using the proportion of playtime that was active as the criterion ^1^.

Predictor	*b*	*b* 95% CI [*LL*, *UL*]	*sr* ^2^	*sr*^2^ 95% CI [*LL*, *UL*]	Fit
(Intercept)	0.46 **	[0.31, 0.61]			
Poster Type	0.09	[−0.07, 0.25]	0.01	[−0.02, 0.03]	
Sex	−0.35 **	[−0.51, −0.18]	0.10	[0.01, 0.18]	
Age	0.07 **	[0.03, 0.12]	0.06	[−0.01, 0.12]	
Poster Type * Sex	0.06	[−0.17, 0.29]	0.00	[−0.01, 0.01]	
					*R*^2^ = 0.185 **
					95% CI [0.07, 0.28]

^1^ A significant *b*-weight indicates the semipartial correlation is also significant. *b* represents unstandardized regression weights. *sr*^2^ represents the semipartial correlation squared. *LL* and *UL* indicate the lower and upper limits of a confidence interval, respectively. * *p* < 0.05, ** *p* < 0.01.

## Data Availability

The data presented in this study are openly available in the Open Science Framework at https://doi.org/10.17605/OSF.IO/VPDT6 (accessed on 3 February 2022).
